# In vivo assessment of safety, biodistribution, and radiation dosimetry of the [^18^F]Me4FDG PET-radiotracer in adults

**DOI:** 10.1186/s13550-024-01098-2

**Published:** 2024-05-15

**Authors:** Barbara Katharina Geist, Juan Carlos Ramirez, Patrick Binder, Holger Einspieler, Harald Ibeschitz, Werner Langsteger, Lukas Nics, Ivo Rausch, Markus Diemling, Antti Sohlberg, Marcus Hacker, Sazan Rasul

**Affiliations:** 1 Division of Nuclear Medicine, Medical University of Vienna, Vienna, Austria; 2 Division of Nuclear Medicine, Unisanitas, Bogota, Cundinamarca, Colombia; 3 Center for Medical Physics and Biomedical Engineering, Medical University of Vienna, Vienna, Austria; 4 Hermes Medical Solutions, Vienna, Austria; 5 Department of Nuclear Medicine, Päijät-Häme Central Hospital, Lahti, Finland; 6 Division of Nuclear Medicine, Department of Biomedical Imaging and Image-guided Therapy, Medical University of Vienna, Waehringer Guertel 18-20, Floor 5L, Vienna, 1090 Austria

**Keywords:** [^18^F], Me4FDG, Tracer biodistribution, Dosimetry, Toxicity

## Abstract

**Background:**

Approaches targeting the sodium-glucose cotransporter (SGLT) could represent a promising future therapeutic strategy for numerous oncological and metabolic diseases. In this study, we evaluated the safety, biodistribution and radiation dosimetry of the glucose analogue positron emission tomography (PET) agent [^18^F] labeled alpha-methyl-4-deoxy-4-[^18^F]fluoro-D-glucopyranoside ([^18^F]Me4FDG) with high sodium-glucose cotransporter and low glucose transporter (GLUT) affinity. For this purpose, five healthy volunteers (1 man, 4 women) underwent multiple whole-body PET/computed tomography (CT) examinations starting with injection and up to 4 h after injection of averaged (2.4 ± 0.1) MBq/kg (range: 2.3–2.5 MBq/kg) administered activity. The PET/CT scans were conducted in 5 separate sessions, blood pressure and temperature were measured, and blood and urine samples were collected before the scans and one hour after injection to assess toxicity. Measurements of [^18^F]Me4FDG radioactivity in organs of interest were determined from the PET/CT scans at 5 time points. Internal dosimetry was performed on voxel level using a fast Monte Carlo approach.

**Results:**

All studied volunteers could well tolerate the [^18^F]Me4FDG and no adverse event was reported. The calculated effective dose was (0.013 ± 0.003) mSv/MBq. The organs with the highest absorbed dose were the kidneys with 0.05 mSv/MBq per kidney. The brain showed almost no uptake. After 60 min, (12 ± 15) % of the administered dose was excreted into the bladder.

**Conclusion:**

Featuring an effective dose of only 0.013 ± 0.003 mSv/MBq and no occurrence of side effects, the glucose analogue [^18^F]Me4FDG seems to be a safe radio-tracer with a favorable biodistribution for PET imaging and also within several consecutive scans.

**Trial registration number:**

NCT03557138, Registered 22 February 2017, https://ichgcp.net/clinical-trials-registry/NCT03557138.

## Introduction

Glucose transport into cells is mainly mediated by two classes of transporters: glucose transporters (GLUTs) [1] and sodium glucose transporters (SGLTs) [2]. In particular, SGLTs, mainly located in the intestine and the kidneys, absorb glucose from food to avoid urinary glucose loss. It was recently found that SGLT genes and proteins are also expressed in various other parts of the body, among others the brain and even in certain cancer cells [3–5] with partially unknown functional relevance. The activity of GLUTs can be studied non-invasively with positron emission tomography (PET) using the glucose analogue2-deoxy-2-[^18^F]fluoro-D–glucose ([^18^F]FDG).

Nevertheless, [^18^F]FDG unfortunately has a very low affinity for SGLTs and thus omits the contribution of SGLTs in glucose related processes. Therefore, a special PET tracer was developed: α-methyl-4-[^18^F]fluoro-4-deoxy-D-glucopyranoside ([^18^F]Me4FDG), which has a high affinity for SGLT1 and SGLT2 and a very low affinity for GLUTs [6]. It does not penetrate the blood brain barrier (BBB) and hence, unpractical for studying SGLT activity in the healthy brain. Still, it was recently found to be a very promising tracer for certain brain tumors [7, 8]. Unlike [^18^F]FDG, the tracer [^18^F]Me4FDG is not phosphorylated but is effectively trapped, particularly in the kidneys, which have not only high SGLT2 but also SGLT1 expression levels [6], as well as in the heart [9] and in certain cancer cell types [5]. Due to its characteristics, this tracer is also of interest for patients suffering from diabetes type 2, where it has already been used in a study conducted at our institution [10]. Although we monitored the general health status and possible effects of toxicity of this tracer in all diabetic subjects during that study, this tracer was never investigated in healthy subject.

Hence, we present here the results of a study to assess the safety, biodistribution and radiation dosimetry of this promising new tracer [^18^F]Me4FDG. It was administered to five healthy volunteers, followed by several dynamic and static PET acquisitions. The study was designed according to guidelines of the European Medicines Agency for conducting a study with healthy volunteers [11] with regards to patient’s health or lifestyle to minimize confounding factors.

## Materials and methods

The study was approved by the Ethics Committee of the Medical University of Vienna (EK: 1899/2018) and by the Federal Office for Safety and Healthcare in Austria (EudraCT No: 2018-002972-42), trial registration number: NCT03557138.

### Subjects

All subjects gave written informed consent and met the inclusion criteria, which were age > 18 and < 75 years, healthy, no previous history of oncological and endocrinological diseases, non-smoker, body mass index (BMI) below 30 kg/m², radiation exposure < 10 mSv within the last 10 years, and written informed consent. Exclusion criteria were any organ dysfunction, claustrophobia, pregnancy, and a positive Covid PCR test. Collectively, five healthy adult subjects (1 male and 4 female) were investigated and met the above-mentioned criteria. Subjects were asked to fast at least 6 h and to empty their bladder immediately before conducting the first PET/CT scan. No further bladder emptying was requested in the first hour of the scanning period.

### [^18^F]Me4FDG radiosynthesis

[^18^F]Me4FDG was prepared in-house following a standardized protocol, using a GE FASTlab synthesizer (GE Healthcare, Boston, MA, USA) with dedicated disposable cassettes on the day of PET imaging. A regular cassette for [^18^F]FDG was used and prepared as follows: The FDG-precursor-vial was removed and exchanged to a 11 mL crimp-vial, filled with a solution of 10 mg GMP compliant precursor for Me4FDG (β-D-Galactopyranoside, methyl, 2,3,6-triacetate 4-trifluoromethanesulfonate, obtained from ABX advanced biochemical compounds GmbH, Radeberg, Germany) in 2 mL acetonitrile. Full radiopharmaceutical quality control according to the monographs of the European Pharmacopoeia (Ph. Eur.) was thoroughly performed prior the release of the tracer and application to the patient. The prepared cassette lost its GMP compliant due to the manipulation but since a radiosynthesis for a subsequent in-house application at the Vienna General Hospital is exempt from any GMP regulation, this route could be taken.

### Examination and safety assessment

**Fig. 1:**
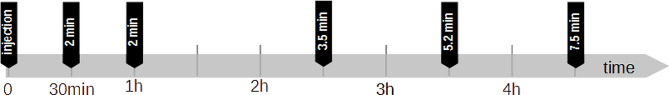
[^18^F]Me4FDG PET scan schedule

A venous cannula was placed in the arm of each subject. Before activity administration, vital parameters were determined by measuring blood pressure, pulse, body temperature. Additionally, one blood sample was drawn for the determination of a full blood count, including kidney, liver and inflammation markers, and one urine sample was collected for the determination of glucose and protein. [^18^F]Me4FDG was administered as an intravenous bolus injection, followed by a flush with 5 mL of saline solution. Before the injection, subjects were placed in a whole-body PET/CT (Biograph Vision Quadra, Siemens Healthineers) with a 106 cm axial PET field of view. The first acquisition started with injected to obtain a 30 min dynamic scan (rebinned into 15 two-minute frames), followed by 4 static images within four hours starting at 60 min, 150 min, 210 min and 270 min pi. The scan time was adjusted for each time point to account for the decay of [^18^F] and was 120 s, 210 s, 312 s and 450 s, respectively. A scheme of the imaging procedure is shown in Fig. 1. All PET images were accompanied by an ultra-low-dose CT scan for attenuation correction purposes (Settings: CAREDose4D with ref tube current 10 mAs and ref. tube potential 100 keV, pitch = 1.5, iterative reconstruction using ADMIRE with strength 3). The PET images were reconstructed using an OP-OSEM algorithm with PSF correction and TOF information with 4 iterations and 5 subsets. Matrix size was 440 × 440 resulting in a 1.65 mm²-pixel size. A 3 mm FWHM Gaussian blurring was applied to the reconstructed images. To evaluate toxicity of [^18^F]Me4FDG, all vital parameters mentioned above were determined after one hour, i.e., a second blood and a second urine sample were obtained, accompanied by a measurement of body temperature and blood pressure. A standard physical examination was performed during the screening visit, any conspicuousness was documented.

### Biodistribution and radiation dosimetry

From the imaging protocol, static whole-body images were reconstructed from 5 time points (Fig. 1). The PET images were used to evaluate the biodistribution of renal cortices, heart and bladder by measuring the tracer concentration as standardized uptake value (SUV).

Radiation dosimetry was performed with Voxel Dosimetry program (HERMES Medical Solutions, Stockholm, Sweden). Briefly, the 5 time points were first registered to a common reference (the first time point) using a 6-parameter rigid registration algorithm followed by a Demons-type [12] non-rigid registration. Registration was performed using CT images and the transformation parameters and deformation fields were later applied also to PET images. After registration voxel level time-activity curves were generated and integrated to obtain voxel level cumulated activities. Since the first scan data were acquired dynamically starting from time zero, a linear activity grow from zero to the activity at the first time point was chosen. From last time point to infinity, a voxel level mono-exponential fit to the last two time points were made, the half-life from the fit was taken and used that after the last time point, whereas the physical half-life was taken if it was shorter. Voxel level photon tracking and absorbed dose map were performed using a fast Monte Carlo algorithm [13]. From the dose map images, volumes of interest (VOIs) were manually delineated from all organs or areas of the body that could be visually distinguished from the background. These were, brain, subcutaneous tissue of abdomen, breasts, colon ileocecal, gluteus maximus, tongue, lungs (average of left and right lung is presented), vertebral bodies L1-L5, spleen, heart, optical nerves (average of left and right optic nerve is presented), pancreas, liver, bladder as well left and right kidneys. In addition, only an ROI (region of interest) was drawn for the abdominal aorta and inferior vena cava, both at the level of the first lumbar vertebra, because it was not possible to follow the entire course of these vessels. The percentage of radiotracer excreted after one hour was assessed from the scan 60 min post injection by calculating the ratio between total bladder uptake and total body uptake.

After intravenous injection of [^18^F]Me4FDG, 5 PET/CT acquisitions were performed, with increasing scanning time from 2 to 7.5 min. The subjects were asked to rest and to void their urinary bladder after one hour.

### Statistics

Statistics in this study were limited to descriptive statistics, calculated with Microsoft Excel 2016. Values are presented as mean value plus/minus one standard deviation (SD). Differences between vital parameter were determined using a student’s T-Test.

## Results

Four of the five participants were female. Patient mean age was (21 ± 7) years and mean BMI was (22 ± 2) kg/m².

### Safety assessment

No adverse events were reported and [^18^F]Me4FDG was well tolerated. All vital parameters determined from blood and urine samples were not altered significantly one hour after the injection, see Table 1.

**Table 1:** Vital and laboratory parameters (full blood count) before and one hour after the intravenous injection of [^18^F]Me4FDG

Parameters	Before injection	1 h after injection	P-value
**Vital parameters**			
^*^BP (systolic/diastolic)	127 ± 10 / 81 ± 6	126 ± 4 / 82 ± 5	0.9 / 0.7
Pulse	74 ± 3	72 ± 8	0.5
Body temperature	36.5 ± 0.5	36.6 ± 0.6	0.8
**Blood parameters**			
Erythrocytes	4.1 ± 0.2	4.1 ± 0.4	0.8
Hemoglobin	12.7 ± 1.3	12.7 ± 1.6	0.8
Hematocrit	37.2 ± 3.1	37.5 ± 4.4	0.8
Thrombocytes	312 ± 48	315 ± 53	0.7
Leucocytes	7.5 ± 1.7	7.6 ± 1.2	0.8
Neutrophils (absolute)	4.6 ± 1.6	4.7 ± 1.0	0.8
Lymphocytes	2.3 ± 0.7	2.3 ± 0.4	0.9
Monocytes	0.5 ± 0.1	0.5 ± 0.1	0.2
Eosinophils	0.1 ± 0.2	0.1 ± 0.1	0.4
Basophiles	0.0 ± 0.1	0.0 ± 0.0	0.4
Creatinine	0.8 ± 0.1	0.8 ± 0.1	0.9
Urea	14 ± 4	14 ± 3	0.3
Uric acid	3.3 ± 1.7	4.1 ± 0.9	1.0
ASAT	24 ± 2	21 ± 4	0.4
ALAT	34 ± 30	30 ± 26	0.8
^$^GGT	13 ± 5	14 ± 5	0.1
C-reactive protein	0.4 ± 0.5	0.4 ± 0.5	0.7
**Urine parameters**			
Inorganic Phosphates	37 ± 55	12 ± 13	0.3
Glucose	10.0 ± 9.1	3.0 ± 2.8	0.3
Protein	0.1 ± 0.1	0.0 ± 0.0	0.1

(^*^) blood pressure; (^$^): gamma-glutamyl transferase. Values are presented as mean value ± one standard deviation, the p-value of the difference was calculated from the student’s T-test

### Biodistribution

**Fig. 2:**
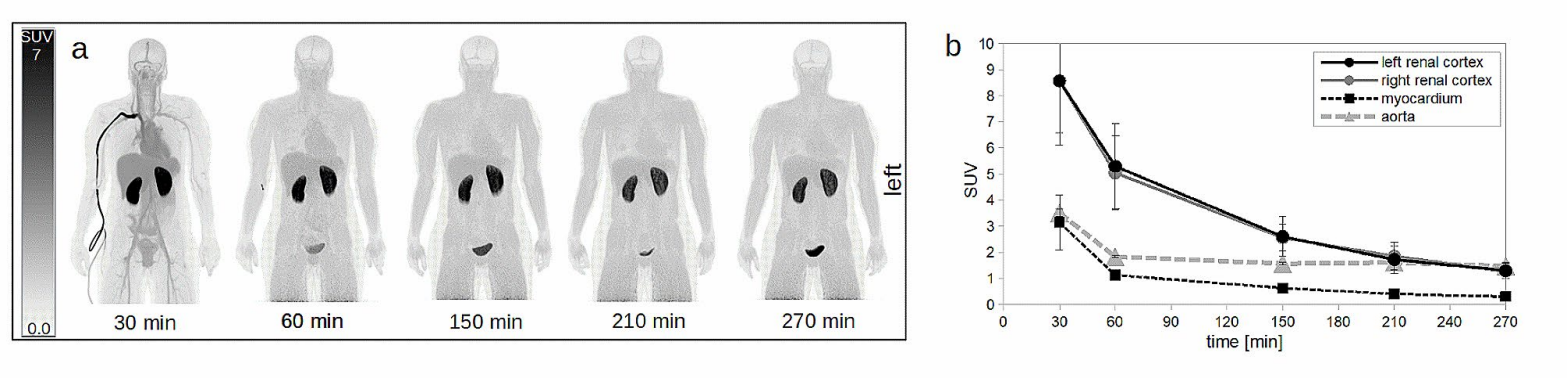
Dynamic series of static images of [^18^F]Me4FDG. **a**: dynamic series of static images of [^18^F]Me4FDG showing the biodistribution of this tracer. The first image represents a sum of the first 30 min. **b**: dynamic uptake behavior, with the renal parenchyma showing the highest uptake at the beginning of the tracer injection. Mean SUV of all referring regions is shown. SUV: standardized uptake value

In average, (2.4 ± 0.1) MBq per kg bodyweight (range: 2.3–2.5 MBq/kg) were injected. Organs showed in general low uptakes, see Fig. 2a, while the renal cortices were clearly visible with a SUV of in average (8.6 ± 2.3) after 30 min (average of all left and right kidneys), which slowly dropped over time until a SUV of (1.3 ± 0.2) after the full examination time of 270 min. All according time activity curves including heart are presented in Fig. 2b.

### Radiation dosimetry

**Fig. 3:**
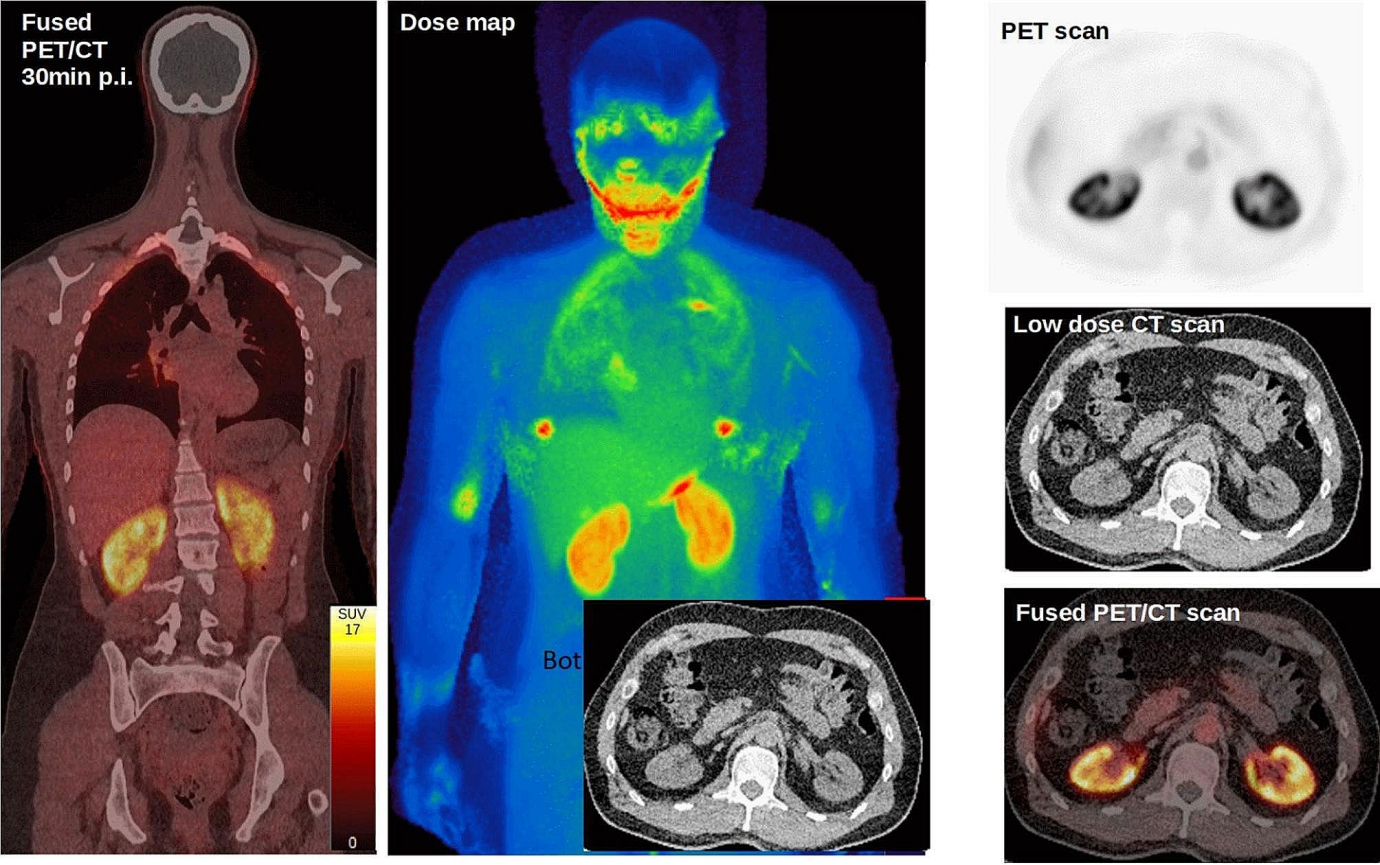
Biodistribution of [^18^F]Me4FDG. Left: Fused PET/CT image of the last scan at 270 min showing the biodistribution of [^18^F]Me4FDG with the highest concentration measured in the renal cortices. Middle: Dose map in units of mSv of the same individual. Right: Transaxial view of the kidneys in the PET images, the low dose CT images as well as the fused scans

In Fig. 3, the dose of [^18^F]Me4FDG from one representative female subject is shown 270 min after injection. The calculated effective dose was in total (0.013 ± 0.003) mSv/MBq. The organs with the highest absorbed dose were the kidneys with 0.05 mSv/MBq per kidney while the brain showed almost no uptake, see Fig. 3; Table 2. After 60 min, 12 ± 15% of the administered dose was excreted into the bladder. For the individual uptake of all organs that had visible tracer uptake, see Table 2.

**Table 2:** Calculated dose in mGy/MBq for all organs presented as mean value plus/minus standard deviation of the 5 investigated individuals

Organs	mGy/MBq
Total brain	0.003 ± 0.001
Subcutaneous tissue – abdominal area	0.009 ± 0.004
Breast (right)	0.013 ± 0.004
Colon ileocecal	0.014 ± 0.005
Gluteus maximus	0.015 ± 0.002
Tongue	0.017 ± 0.003
Lungs	0.019 ± 0.005
vertebral bodies L1-L5	0.019 ± 0.005
Spleen	0.023 ± 0.006
Heart	0.024 ± 0.005
Optic Nerve	0.026 ± 0.005
Abdominal Aorta (ROI)	0.026 ± 0.006
Pancreas	0.027 ± 0.007
Inferior vena cava	0.027 ± 0.005
Liver	0.028 ± 0.006
Bladder	0.038 ± 0.014
Kidney left	0.053 ± 0.015
Kidney right	0.055 ± 0.014

ROI: Region of interest

## Discussion

[^18^F]Me4FDG is a promising new radiopharmaceutical relevant for patients with certain cancer types and for patients with diabetes mellitus.

Herein, we investigated clinical safety, biodistribution and radiation dosimetry of [^18^F]Me4FDG in 5 healthy adult volunteers with no history of oncologic or endocrinologic diseases While this tracer demonstrated no side effects in patients with type 2 diabetes in a 3-month longitudinal study [10], to our knowledge it has not yet been systematically studied in healthy volunteers.

Since [^18^F]Me4FDG is a glucose analogue like [^18^F]FDG, we decided to apply a standard activity dose as used for [^18^F]FDG examinations. A mean effective dose of 0.013 mSv/MBq was measured, which is similar to the routinely used glucose analogue [^18^F]FDG [13, 14]. However, new generations of PET/CT imaging scanners and recent efforts have indicated that a lower [^18^F]FDG dose is preferable [15, 16], and thus the [^18^F]Me4FDG dose can likely be reduced as well. Moreover, from the findings of the study, it is clear that [^18^F]Me4FDG was very well tolerated by the volunteers, as we did not detect any significant changes in the measured vital and laboratory parameters before and one hour after the intravenous injection of this tracer. In addition, no adverse drug reaction to the tracer was experienced by the subjects during the entire study period.

Concerning the biodistribution of this tracer and since Me4FDG does not cross the blood-brain barrier [6], it is plausible that no uptake of the tracer could be measured in the brain. In general, various organ uptakes were observed, and as expected, the highest [^18^F]Me4FDG uptake was found in the renal cortex. In this region, both SGLT1 and SGLT2 are strongly expressed [3, 17] and this tracer has a high affinity for these two cotransporters [6]. In addition, SGLT1 is broadly expressed in many other organs such as the liver and heart [18] which may explain the apparent tracer uptake in these organs in the individuals we studied, see Table 2; Fig. 3.

While there are certain rare cancers [3–5] that warrant the application of this diagnostic tracer, it may also be of interest to utilize this tracer to understand the effects and consequences of SGLT1 inhibition therapies on the heart, where SGLT1 expression is associated with oxidative stress, inflammation and mitochondrial dysfunction [9]. As evidenced by the aforementioned study involving type 2 diabetes mellitus [10], Me4FDG may be able to be potentially employed to investigate the contribution of the renal system to obesity-related diseases, as Me4FDG is able to determine the threshold of renal glucose reabsorption, a process carried out exclusively by the two transporters SGLT1 and SGLT2.

Moreover, since SGLT2 is exclusively expressed in the renal cortex, which - as we have demonstrated - results in excessive tracer uptake in the renal cortex, Me4FDG could also be of interest for theranostic applications where only the kidneys need to be irradiated, e.g. in the case of kidney cancer. Also recent findings in rodents suggest to use this tracer for renal examinations [19]. In particular, it was shown that renal uptake can be altered due to SGLT inhibition treatments, which was also already implied by our study [10]. Moreover, the animal data found that Me4FDG can be used to determine the effects of SGLT inhibition in the digestive tract [20], suggested to be used as patient selection tool for diabetic patients designated for SGLT inhibition therapy.

Additionally, from the outcome of our earlier study, we are aware that the degree of washout of this tracer from the blood pool is comparable to that of [^18^F]FDG, allowing static PET acquisition 60 min after tracer injection without expecting a high degree of tracer circulation in the blood pool [10].

## Conclusion

From the results of this study, we concluded that using an administered activity of averaged 2.4 MBq [^18^F]Me4FDG per kilogram body weight, no adverse or clinically detectable pharmacologic effects were found in any of the 5 healthy voluntary subjects, scanned in a period of four hours. A mean effective dose of only 0.013 ± 0.003 mSv/MBq was determined. The biodistribution and radiation dosimetry of the novel glucose analogue [^18^F]Me4FDG are favorable for PET imaging, and it is a safe tracer, also within several consecutive scans, which is relevant for potential applications in cancer imaging.

## Data Availability

The datasets used and analyzed during the current study are available from the corresponding author on reasonable request.
